# Abstract of the Proceedings of the Buffalo Medical Association

**Published:** 1872-07

**Authors:** L. F. Harvey

**Affiliations:** Sec.


ART. II.—*Abstract of the Proceedings of the Buffalo Medical Association. May 7th,* 1872.

Members present: Drs. Cronyn, Wycoff, Hauenstein, Little, Diehl, Strong, Bartlett, Sloan, Barnes, Phelps, Johnson and Ring.

Dr. Cronyn in the chair. On motion the reading of the minutes was dispensed with.

Dr. Hauenstein moved that the proceedings of one meeting be read at the meeting, after which they could be published. Carried.

Dr. Strong as chairman of the committee to present a memorial to the city authorities, asking for a report of the board of health, reported that such a memorial had been presented, and they had the assertion of Dr. Stork as to its propriety, and he in this cursory manner promised to use his influence to have such a report made for 1871, and the committee think it will be published.

Dr. Wyckoff moved that the report be accepted and that the committee be discharged. Adopted.

Dr. Cronyn.—The prevalence and fatal nature of what is called indifferently, cerebro-spinal meningitis or spotted fever, attracts the attention of the profession just now, more especially as there are very discordant views with regard to its pathology and treatment. Without entering into the opinions of others as to what they think it and why, I will very briefly state my own. I consider it accurately described by Trousseau in his "cerebro-spinal typhoid." The mode of attack is very generally the same in all cases, though some are not so violent as others; e.g. in one case, vomitting and purging with violent headache and opisthotonos are present from the beginning, lasting one or two days; in another, vomiting, headache and excruciating pain of back, resembling the accession of variola, are present for the first twenty-four hours. Again only violent headache and general malaise for the first thirty-six hours, when severer symptoms set in as opisthotonos, difficulty in deglutition, dry brown tongue, delirium and if the bowels were loose at first, they become constipated, if constipated, they become loose, the discharges having a *true* typhoid character. In all the cases that I have seen, three distinct stages could be marked: the first and shortest being one of great excitement and pain; the second of coma or semi-coma and less delirium, with the cervical muscles in painful contraction; and third great exhaustion, accompanied with gastro-intestinal epithelial exfoliation and diarrhœa.

In this cursory description of the disease as I see it, it may be asked how I can account for the suddenness of attack and of death in many of the cases, as they do not pursue the ordinary course of typhoid, nor do they seem to have the same etiology, and they do very closely correspond to Niemeyer's description of cerebro-spinal meningitis. Now I believe that many of the suddenly fatal cases reported were malignant variola, and if they were not, a sudden and fatal termination with a form of typhoid, which I think prevails, is not uncommon. Why, in the ataxic form of Trousseau, many features of which are common to that under discussion, patients are "killed as if by a thunderbolt! Thus therefore there need be no question as to its typhoid character, because in this particular, of sudden death, it differs from the ordinary course of that fever. In its etiology, to my mind, the resemblance to typhoid is even more marked, as in every instance which came so far under my notice, it was distinctly traceable to the ingesta, whether of food or water, but of the latter more particularly; and where it failed to correspond with the elaborate description of Niemeyer in the only typical case of which an examination post mortem was had, no *lesion* of the brain or spinal cord was found.

The indication for treatment is too plainly deducable from this view of the pathology of the disease and need not occupy our time.

Dr. Strong said he was gratified to have Dr. Cronyn's observations confirm his own conclusions. He considered the term *cerebro-spinal meningitis,* as applied to the disease, somewhat prevalent this winter and spring—a *misnomer,* certainly as applied to most cases. He could not say how it came to be considered a meningitis,—except perhaps it be the occipital and cervical pain together with the somewhat distinctive contraction of the posterior muscles of the neck—neither of which can be taken as conclusive of meningeal inflammation.

In my observation, so far as the pain is concerned, it is far more neuralgic than inflammatory, and as to the muscular contraction, of course that does not imply inflammation.

In one of my most typical adult cases, while there was the usual occipital and cervical pain, by far the severest suffering was from pain in the *calves of the legs* and especially the *poplitial region*; there it was simply intense, extorting the sharpest outcries from one little accustomed to give vent to her feelings. She spoke of it, as feeling as if the *bone would burst.* It was paroxysmal, not attended with swelling, and was clearly neuralgic. It only yielded to grain doses of morphia, which also relieved the pain in head and neck. This, with quinine, iron and gin, to speak briefly, was the sum of treatment, under which the disease after five or six days culminated.

What else, whether we regard the symptoms or treatment, could it (the pain) have been, than an aggravated neuralgia? In another case of a boy of three and a half years, with similar pain in head and neck and with still more marked rigidity and contraction of pos erior muscles of the neck, the symptoms yielded to substantially similar treatment.

Indeed this treatment essentially,—though from being out of the city much of the time I have not seen a very large number of cases,—has been effectual in subduing the disease in most of my cases.

The treatment, to speak of nothing farther, to my mind is conclusive against the nature of the affection being regarded as a meningitis. The conclusion seems legitimate and logical, that this designation for such cases, is a misnomer. But I go farther and say that so to regard it is a pernicious delusion, for the obvious reason, that it suggests and prompts on the one hand, to active measures, that in the nature of the case must be pernicious and on the other hand ignores, those, the omission of which is but little less so. So to regard it therefore, leads us to the twofold offenses of commission and omission. If this view is tenable, why longer cling to the misleading designation, cerebro-spinal meningitis?

The term spotted fever, has the merit of not being positively mischievously misleading. At the same time it seems to me a rather stupid term for this disease in as much as the spots called for, are in my experience comparatively rarely seen, and never after the first day or two. It may have to be endured till we devise a better one. I shall be glad if *The Buffalo Medical Association* can have the honor of leading off, not only in discarding a mischievous designation, but in suggesting one, which shall indicate the nature and seat of this grave affection. But it is asked, how account for the cases, that terminate in coma and death either suddenly or more remotely?

I would by no means be understood, as contending that such cases may not eventuate in meningitis or other cerebral and spinal lesions, but is not an analogous termination often seen in typhus, typhoid and even in pernicious intermittent and scarlet fever, not to mention other affections. And have we ever thought of these as essentially brain affections? And this brings me to consider briefly the positive nature and source of this somewhat unique affection.

And here I must confess myself as convinced of Trousseau's correctness, as I understand him, in regarding the disease as a peculiar and essential fever, superinduced upon some morbific impression made primarily upon the great sympathitic and ganglionic system of nerves, and radiating therefrom to the various organs. Upon this theory of the affection it seems to me, can most, if not all, of the phenomena observed, be most rationally explained.

As to its causation, contrary to the popular impression, I have seen nothing to support the idea that it was infectious, or communicable, by one person to another. All the facts that I have observed controverted the notion. True, it often attacks several members of the same family, but never under my observation in circumstances to necessitate the conclusion that one took it from another. On the contrary the clinical facts, combined to support the idea, that all who were seized in the same household, were so seized from exposure to some morbific agency which was common to all attacked, some disease-engendering principle inhaled or ingested (probably the latter), to which those attacked were alike exposed. I say more likely ingested than inhaled, because in the cases I have observed, the premises and surroundings were faultlessly clean, the rooms well ventilated, the air indicating no offensive odor or impurity to the sense of smell. Moreover, the season in which they occurred, the ground and all external sources of miasmata being frozen, and thus innocuous, the premises being often in most elevated and airy localities, to my mind point less to an atmospherical source than to some other, unless indeed, it be some thermometric, barometric, hygrometric or electrical change, of whose conditions and their consequences we have little positive knowledge. Unless. I say, it be some such agency, not perceptible to the senses, it seems to me more rational to look to the ingesta for the proximate cause. Now the inquiry, *which* ingesta, whether liquid or solid, and what of either, can be the cause, of course leaves us with a wide field for speculation and hypothesis.

Diseased cereals suggest one, with some facts to support it. Various other sources are indicated by others. For my own part, I am inclined to suspect impure water as playing a leading part in the causation. I have no facts that are at all conclusive on the subject and would like to suggest it for consideration, more as hypothetical than aught else. But if it be the fact, as has been alleged that most of the cases have occurred in families, supplied by well-water from surface saturation, it strikes me as somewhat significant and suggestive. I believe most of the families in which I have seen the disease have depended upon this source for their water, and I shall be pleased to hear from other gentlemen present, if their observations either tally with or differ from my own.

DR. CRONYN.—I do not deny the existence of cerebro-spinal meningitis, but the present disease is not that affection. Have found the disease prevalent in some localities where families have used wells in common. In one locality on Tenth street I had five cases. Seems to be most prevalent where the water is sulphurous or saline. Would throw out the idea that water has to do with the disease for reflection.

DR. HAUENSTEIN.—I rise simply for the purpose of being on record as one having the opinion that the so called cerebro-spinal meningitis, which now prevails as an epidemic in this city, is a disease *sui generis* and, according to my limited observation of it, the disease seems to me so peculiar in its character and so different from any disease I have seen, that I am at a loss to detect any identity between it and the different forms of typhoid fever, which I have observed for the last twenty-five years or more. Its mode of attack is sudden, differing in that from typhoid fever as a rule When once established, its more prominent symptoms show, certainly, marked peculiarities. Its course from a few days, or even less, to a few months is a remarkable feature of cerebro-spinal meningitis. I have in no case observed marked tympanitis and in no case persistent diarrhea.

As regards treatment I have nothing new to suggest. I have administered anodynes and in one case I thought I saw marked improvement from the application of a blister to the nape of the neck and subsequent dressings with an ointment containing extract of belladonna. From the use of bromide of potassium and quinine I have not derived very satisfactory results. A case of the disease in question came into my hands, after having been treated for three weeks by an irregular practitioner, and died after the eleventh week of illness. Not only its neck was rigid, but its abdomen also and the lower extremities were rigidly flexed, the body extremely emaciated. I have also seen two cases occurring in the aged, one a woman of 60 years of age, and another a woman of 67 years of age, both died.

Dr. Bartlett had listened with great interest to the remarks of the gentlemen who had addressed the association. Considered the identity of the present epidemic with typhoid fever not well established. Believed food and drink had but slight, if any, influence in the causation of the disease. Referred it to atmospheric or electric influences, but admitted the whole subject was wrapped in mystery.

His observation covered thirty-one cases of the disease. Twenty-seven of these had occurred in his own practice, the remaining four in that of medical friends. Mortality, so far as known, six. Of the surviving patients twenty may be considered recovered. One other recovered except the sense of hearing. Of those dying two had died within twenty-four hours of attack, one on the 4th day, one on the 8th, and one on the 9th. All of his cases had opisthotonos in greater or less degree; contrary to the observation of others, diarrhea was not present in more than two or three cases. Cathartics and laxatives were required in all the others. Very few of the patients had tympany or tenderness on pressure of the abdomen. Functions of the mind more or less disturbed in all.

Treatment in all but the first three or four cases was as follows: Full dose of castor oil to move the bowels; if not promptly effective then injection of castor oil and turpentine; the "hot pack," by enveloping the patient, except the head, in a sheet wrung from hot water and applied as hot as it could be borne; over this a blanket and external to that other blankets or bedding; to the head a napkin wrung from cold water. Patient allowed hot or cold drinks *ad libitum*. The free use of spiritus ætheris nitrosum, potassæ acitatis, or other diuretic.

Profuse perspiration comes on generally in from fifteen to thirty minutes, and the patient was kept perspiring from four to six hours; then removed and warmly dressed. The effect seemed to be most salutary and beneficial as a preliminary treatment.

Though not convinced of its utility, counter-irritation to the spine and abdomen has been practiced in all the cases: the embrocation more usually employed is as follows:

℞ Ol. Terebinthinæ,
Tr. Aconite rad.,
Tr. Iodine, Co., aa ℥ss.
Lini Saponis, q. s. ad. ℥vj.
M.

S. Apply every three or four hours along the entire spine. Internally, the bromides, at times combined with chloral. At later stages Iodide Potass., in three or four grain doses every four or six hours. Any increase of cerebral distress to be met by hot fomentations to the abdomen and moderate use of opium. The large and repeated doses recommended by some, though giving temporary relief, did not seem to have a favorable effect upon the course of the malady, especially contra-indicated when pupils were contracted In evidence of the value of the "hot pack," would cite two cases.

Was called April 4th to see the child of Mr. B., residing at No. 27 7th street, aged about thirteen years. Found patient with cerebro-spinal meningitis, had been ill for two days, but without medical advice. Predicted a fatal issue, all the worst symptoms being present. Used counter-irritation to spine. Cold to head, opiates and cathartics. Patient worse next day and evidently failing; died on Sunday evening, the second day after treatment, and fourth day of attack. Instructed the parents in the use of the hot pack, and directed its employment should another case occur.

Four days after death of patient referred to, was summoned in haste to attend a child of eight years in same family, with same symptoms. The hot pack had been promptly employed and vomiting and pain speedily relieved. Child was sleeping when he arrived. Advised hot applications continued with diuretics. Six hours later a child of five years was taken with same symptoms in a very severe degree; same treatment. Saw both patients in the evening, eight hours after his first call, and four hours after attack of younger child. Twenty-four hours after, younger child was permitted to be dressed and required no further medication. The older child was kept in bed three days and was fully recovered. Instances like these might be multiplied, but the procedure must commend itself to favorable consideration without further comments.

DR. PHELPS.—Had some cases on 5th street; in the house there is no drainage; water in the cellar. Family use pump water; had not thought that the water might be a cause.

Five years ago a healthy family moved into a house in the lower part of the city; had five children sick in a few months, on looking for the cause, found the sewer broken and its contents were running into the well.

DR. CRONYN.—There is diarrhea in second stage in nine cases out of ten, and is controlled by opium. The water in wells is not always the same; more sulphur in a pint of water some days than others. In London, sewers and the atmosphere have an effect on wells.

DR. STRONG.—I cannot conceive how miasmata from external sources, in winter and early spring in this latitude, can be a very fruitful source of disease. We all know, I suppose, that freezing is only less destructive than heat to boiling point, to vegetable and animal miasmata, and that if not absolutely annihilating, it certainly suspends their active potency as morbific agents, while the congelation continues, so that I am inclined to put the amount of disease, originating from this source in freezing weather, at a pretty small minimum. But beyond much doubt, conditions of the atmosphere, pertaining to its temperature, weight, humidity and electricty, play a quite important, though as yet largely unrecognized, part in the production of disease.

But I can readily understand how the water of surface wells could be a prolific source of disease, especially in a season of protracted slight rain-fall as was the past autumn and winter.

The water of wells not being replenished, of course, by use and evaporation progressively diminishes; and just in the ratio of its decrease, its impurities, (more or less of which are incident to all undistilled water, albeit, when water is abundant and often changed, they are so widely diffused as to be innoxious), are proportionately increased. A glass of water from the same well may thus contain two, three or four times the amount of impurities at one time that it does at another; due entirely to this process of elimination and concentration.

It has been notorious the past winter and spring, throughout this city and over a large tract of country, that the wells have been very low and in very many cases wholly dried up. And it really does not seem to demand a very lively imagination, to rationally infer that this condition of an essential to life, ingested by many every hour, and by all with greater or less frequency, may have proved noxious and disease-engendering.

It has been a very common remark that the water, on which the citizens are wont to rely, has had an unnatural, often disagreeable taste.

Moreover, many of us are not quite disposed to acquit the Niagara water of all responsibility in the matter of sickness, especially at certain periods of every winter and spring, when it is a shocking abuse of the King's English, to call it any thing like pure.

I believe the better opinion of the profession is getting to be, that typhoid fever is often, if not uniformly generated by morbific principles ingested. Can it be any thing very illogical, to attribute this disease termed by so acute an observer as Trousseau a species of typhoid, to a source so analogous to that of typhoid as I am disposed to claim for it.

Dr. Cronyn.—In the last epidemic there was more true cerebro-spinal meningitis.

Dr. Phelps.—It was a very dry season.

Dr. Ring.—Have seen this disease only within past three weeks. Cannot speak of water as a cause. My cases have been near York street, in high dry places mostly. There is not so much here as at Attica.

My cases have been of a varied character. One a boy of thirteen had violent convulsions, could neither hear, see, or speak when first seen; a few hours of paroxysm, was followed by violent pain in the head; for two weeks saw objects double; during the third week had incontinence of urine. Another, a girl of six years, had fever and chill, convulsions, pulse feeble and weak, stupor as in malignant fever, used stimulants, dry heat, mustard. Case proved fatal in three hours. Others taken with more fever and pain in head.

In one child symptoms were very mild for a few days, then convulsions set in and death very soon followed. I am inclined to rely on morphine and quinine. Need stimulants and heat in bad cases. In mild cases gave opium, quinine, ipecac, in one case gave carb. ammonia. Keep the bowels open if possible. Thought it resembled typhus fever from reading, after seeing it, changed my mind.

*Prevailing diseases,* cerebro-spinal meningitis, pneumonia, whooping cough, and small pox. On motion adjourned.

L. F. HARVEY, Sec.

